# Topographic Relationship between Telangiectasia and Cone Mosaic Disruption in Macular Telangiectasia Type 2

**DOI:** 10.3390/jcm9103149

**Published:** 2020-09-29

**Authors:** Roya Zandi, Jessica Song, Paul S. Micevych, Amani A. Fawzi

**Affiliations:** Department of Ophthalmology, Feinberg School of Medicine, Northwestern University, Chicago, IL 60611, USA; roya.zandi@northwestern.edu (R.Z.); jessica.song@northwestern.edu (J.S.); paulmicevych@gmail.com (P.S.M.)

**Keywords:** optical coherence tomography angiography, optical coherence tomography, adaptive optics scanning laser ophthalmoscopy, photoreceptors, macular telangiectasia, retinal imaging analysis

## Abstract

In this cross-sectional observational study, we investigated the relationship between photoreceptor layer disruption and telangiectasia in patients diagnosed with early stage macular telangiectasia type 2 (MacTel). A total of 31 eyes (17 patients) with MacTel were imaged with adaptive optics scanning laser ophthalmoscopy (AOSLO) and optical coherence tomography angiography (OCTA). Confocal AOSLO was used to visualize dark regions of nonwaveguiding outer segments, which we refer to as “photoreceptor lesions”. En-face OCTA images of the deep capillary plexus (DCP) were used in conjunction with confocal AOSLO to evaluate the topographic relationship between areas of capillary telangiectasias and photoreceptor lesions. Among seven eyes with early stage MacTel (stage 0–2 based on OCT), we identified ten photoreceptor lesions, all of which were located within parafoveal quadrants containing DCP telangiectasia on OCTA. Seven of the lesions corresponded to the intact ellipsoid zone on spectral-domain OCT (SD-OCT), and three of these also corresponded to the intact interdigitation zone. This work demonstrates a topographic relationship between AOSLO photoreceptor lesions and DCP telangiectasias, and it also suggests that these lesions with normal SD-OCT appearance may represent areas of photoreceptors at risk for dysfunction. Thus, confocal AOSLO may have a meaningful role in detecting early photoreceptor abnormalities in eyes with MacTel.

## 1. Introduction

Macular telangiectasia type 2 (MacTel) is an acquired, bilateral degenerative retinal disease that leads to progressive loss of vision. It is characterized by juxtafoveolar telangiectatic capillaries, right-angled venules, loss of retinal transparency, and ellipsoid zone (EZ) loss on spectral-domain optical coherence tomography (SD-OCT) [1,2,3]. Over the last few decades, the basic understanding of the pathophysiology of MacTel has been transformed largely via histopathology studies [4,5,6] and OCT angiography (OCTA) imaging [7,8,9,10]. The pathophysiology of this rare entity is now thought to be related to complex neurovascular dysfunction—namely the loss of Muller cells leading to photoreceptor dysfunction and reactive vascular changes in the outer retina [4,6].

Our recent work on MacTel has shown a strong topographic relationship between telangiectatic vessels in the deep capillary plexus (DCP) on OCTA and EZ loss on SD-OCT [11]. We next wondered whether the telangiectasia that is seen in earlier stages of Mactel, before the appearance of EZ loss, could be related to subclinical photoreceptor dysfunction. The current study seeks to further explore this relationship by investigating the integrity of the photoreceptor layer in eyes with very early stage MacTel, using adaptive optics scanning laser ophthalmoscopy (AOSLO), which provides high resolution images of the intricate photoreceptor microstructures [12,13].

AOSLO has previously been used to study the photoreceptor architecture in MacTel by evaluating the dark regions of hyporeflectivity related to presumed nonwaveguiding photoreceptors [14,15,16,17]. Studies using AOSLO can visualize the earliest subclinical disruption to photoreceptors and therefore bring us closer to understanding the progression of these degenerative changes. Recognizing the neural basis in the reactive vascular changes in this disease has profound implications for the treatment strategies [18,19]. In this work, we hypothesized that areas of telangiectatic vessels in the DCP in early stages of MacTel are closely related to zones of cone mosaic disruption, which cannot be visualized on standard SD-OCT imaging. The results reported herein demonstrate the utility of AOSLO to better understand the pathophysiology of retinal diseases such as MacTel as well as to identify the earliest signs of photoreceptor abnormalities that might otherwise be missed using standard imaging modalities.

## 2. Experimental Section

### 2.1. Subjects

This was an observational, cross-sectional study of 17 patients (31 eyes) diagnosed with MacTel between March 2016 and June 2019 in the Department of Ophthalmology at Northwestern University in Chicago, Illinois. The study (STU00200305) was approved by the Institutional Review Board of Northwestern University, following the tenets of the Declaration of Helsinki. Patients were included in the study after obtaining written informed consent.

Patients were referred to our institution by outside providers for evaluation of retinal telangiectasias and consideration of MacTel trials. The study population was nonfamilial. Each patient underwent a full ophthalmologic examination and multimodal imaging, including color fundus photography (Topcon America, Oakland, NJ, USA), fundus autofluorescence (FAF) (Heidelberg Engineering, Heidelberg, Germany), fundus fluorescein angiography (FFA) (Topcon America, Oakland, NJ, USA), optical coherence tomography angiography (OCTA) (Optovue Inc., Fremont, CA, USA), spectral-domain optical coherence tomography (SD-OCT) (Optovue Inc., Fremont, CA, USA), and adaptive optics scanning laser ophthalmoscopy (AOSLO) (Boston Micromachines Corporation, Boston, MA, USA). A MacTel diagnosis was determined based on characteristic fundoscopic findings and FFA patterns. Eyes were staged using the recent OCT criteria proposed by the Chew et al. classification [20], which take into account the location of EZ loss, presence and location of macular pigmentary deposits, presence of OCT hyper-reflective foci, and presence of subretinal neovascularization. Only early stage MacTel (stage 0–2) eyes were ultimately included in the study.

### 2.2. Optical Coherence Tomography Angiography Imaging Protocol

Images were obtained using the RTVue-XR Avanti system (Optovue Inc., Fremont, CA, USA)with split-spectrum amplitude-decorrelation angiography (SSADA) software (version 2017.1.0.151) [21]. The instrument uses an A-scan rate of 70,000 scans per second and a light source centered at 840 nm with a bandwidth of 45 nm. Two consecutive B-scans (M-B frames), each containing 304 A-scans, were captured and SSADA was used to generate angiographic flow information.

### 2.3. En-Face OCTA Analysis

En-face OCTA images of the superficial capillary plexus (SCP) and deep capillary plexus (DCP) were exported from the RTVue-XR Avanti software using default segmentation parameters. The SCP segmentation is from the inner limiting membrane (ILM) to 55 μm above the inner plexiform layer (IPL). The DCP segmentation is 6 μm above the IPL to 50 μm below the IPL. En-face structural OCT images of the EZ were segmented manually by adjusting the default choriocapillaris parameters to center onto the EZ. The 3 × 3 mm en-face images were imported into PowerPoint (version 16.0, Microsoft, Redmond, WA, USA) for image analysis.

To identify telangiectatic vessels in the DCP, two masked observers (R.Z. and P.S.M.) independently traced vessels that appeared nontapering and dilated using the previously described protocol [11]. All en-face DCP images were cross-referenced with the en-face SCP images to exclude any superficial vessel related projection artifacts.

### 2.4. Evaluation of EZ and IZ Integrity

SD-OCT B-scans from the RTVue-XR Avanti system were used to assess the integrity of the EZ and interdigitation zone (IZ). The scans were inspected for attenuation or disruption of the EZ and IZ as well as any changes to the external limiting membrane. A thorough evaluation of the SD-OCT B-scans for each patient was performed, and areas of EZ loss were recorded.

### 2.5. Adaptive Optics Scanning Laser Ophthalmoscopy Imaging Protocol

Imaging was performed using the Apaeros LF retinal imaging system system (Boston Micromachines Corporation, Boston, MA, USA) based on the optical design of Dubra and Sulai [22]. As previously described [23], the instrument uses a 97-actuator AlpAO DM (AlpAO SAS, Montbonnot, France) with 25 μm of stroke for wavefront correction. Two superluminescent diode light sources are used. An imaging superluminescent diode is centered at 790 nm with a bandwidth of 15 nm, and a wavefront sensing superluminescent diode is centered at 850 nm with a bandwidth of 20 nm. The approximate combined power at the eye is 130 μW.

Confocal en-face AOSLO images, measuring 1.0° × 1.18°, 1.5° × 1.77°, or 3.0° × 3.54°, were obtained in the macula starting from the fovea and extending outwards. A step of 0.5° was used between image sequences to ensure adequate overlap of images and to facilitate montaging.

### 2.6. Adaptive Optics Image Analysis and Montage Generation

Raw confocal AOSLO image sequences were imported into ImageJ (version 1.52j, National Institutes of Health, Bethesda, MD, USA) where representative individual images were selected. Images were selected on the basis of minimal distortion (local shear and compression/expansion) [24] and imported into PowerPoint for analysis.

Selected images were then brightness- and contrast-adjusted to closely match the en-face structural OCT of the EZ, which was used as a guide for manual montaging. 3.0° × 3.54° AOSLO images were stitched together using the en-face structural OCT as a guide. The higher resolution 1.0° × 1.18° and/or 1.5° × 1.77° AOSLO images were then overlaid on the montage created from 3.0° × 3.54° images to create higher resolution montages revealing the outer segment cone mosaic.

For all patients, montages were then analyzed by two masked graders (R.Z. and J.S.) to identify dark regions, previously referred to in the literature as lesions (areas of weakly reflecting or absent cone mosaic on AOSLO) [15]. In this work, lesions were defined as discrete, confluent areas of hyporeflectivity, suggestive of morphologic disruption of the outer segments and/or disturbance of the refractive index of photoreceptor outer segments [25]. We selected areas with the greatest apparent hyporeflectivity, at least 100 μm in diameter, and those that had adjacent areas of normal, hyperreflective outer segments. Areas at the periphery of the montage, the central fovea, and shadows of superficial vessels were not considered. Inclusion and exclusion of lesions was again validated by image analysis using ImageJ. Automatic thresholding with mean method was used to confirm the areas previously chosen as lesions (Figure S1). Only one image required further thresholding to visualize the chosen lesion (Figure S1F).

### 2.7. Image Overlays

PowerPoint was used to generate multimodality image overlays to compare the en-face structural OCT images of the EZ and AOSLO montages. To analyze the AOSLO montages, image overlays of the montage and en-face structural OCT were used. This enabled cross-referencing between images and corresponding SD-OCT B-scans for evaluation of the outer retina.

## 3. Results

**Figure 1:**
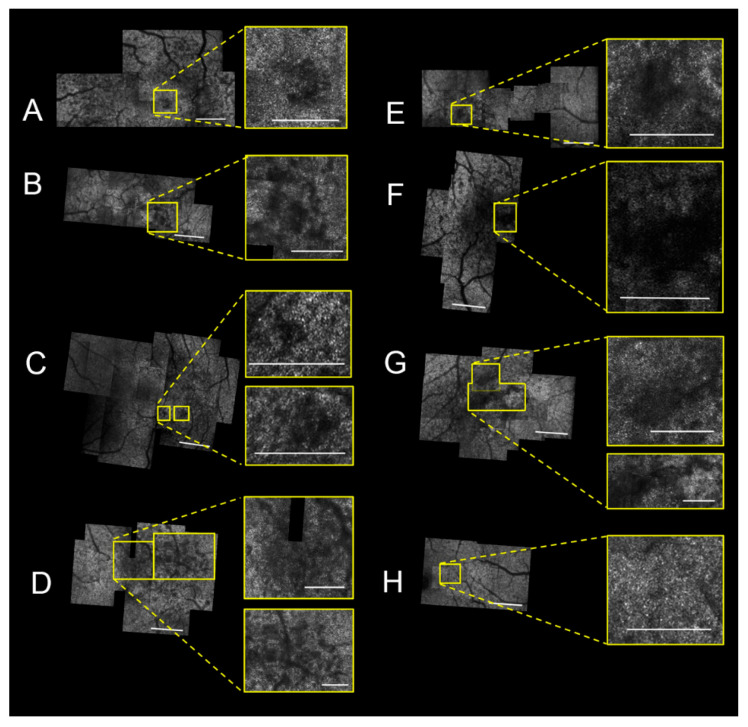
Adaptive optics scanning laser ophthalmoscopy (AOSLO) montage of each eye with macular telangiectasia type 2 (MacTel) reveals photoreceptor lesions. Subjects 1–8 (**A**–**H**). In montages **A**–**G**, 1–2 lesions were identified and magnified (inset). No lesions were identified in (**H**). Montage and inset scale bars represent 500 μm and 250 μm, respectively.

Of the 31 eyes examined and graded based on the Chew et al. OCT classification [20], nine eyes (nine patients) met criteria for stage 0–2 MacTel. One eye was excluded for limited number and poor quality of AOSLO images. Patient characteristics for the eight eyes are summarized in Table 1. The mean patient age was 57 and mean visual acuity was 20/25. A compilation of the AOSLO montages with photoreceptor lesions (yellow box) for each eye is shown in Figure 1. The excluded eye in these patients met criteria for stage 3 or more advanced.

**Figure 2:**
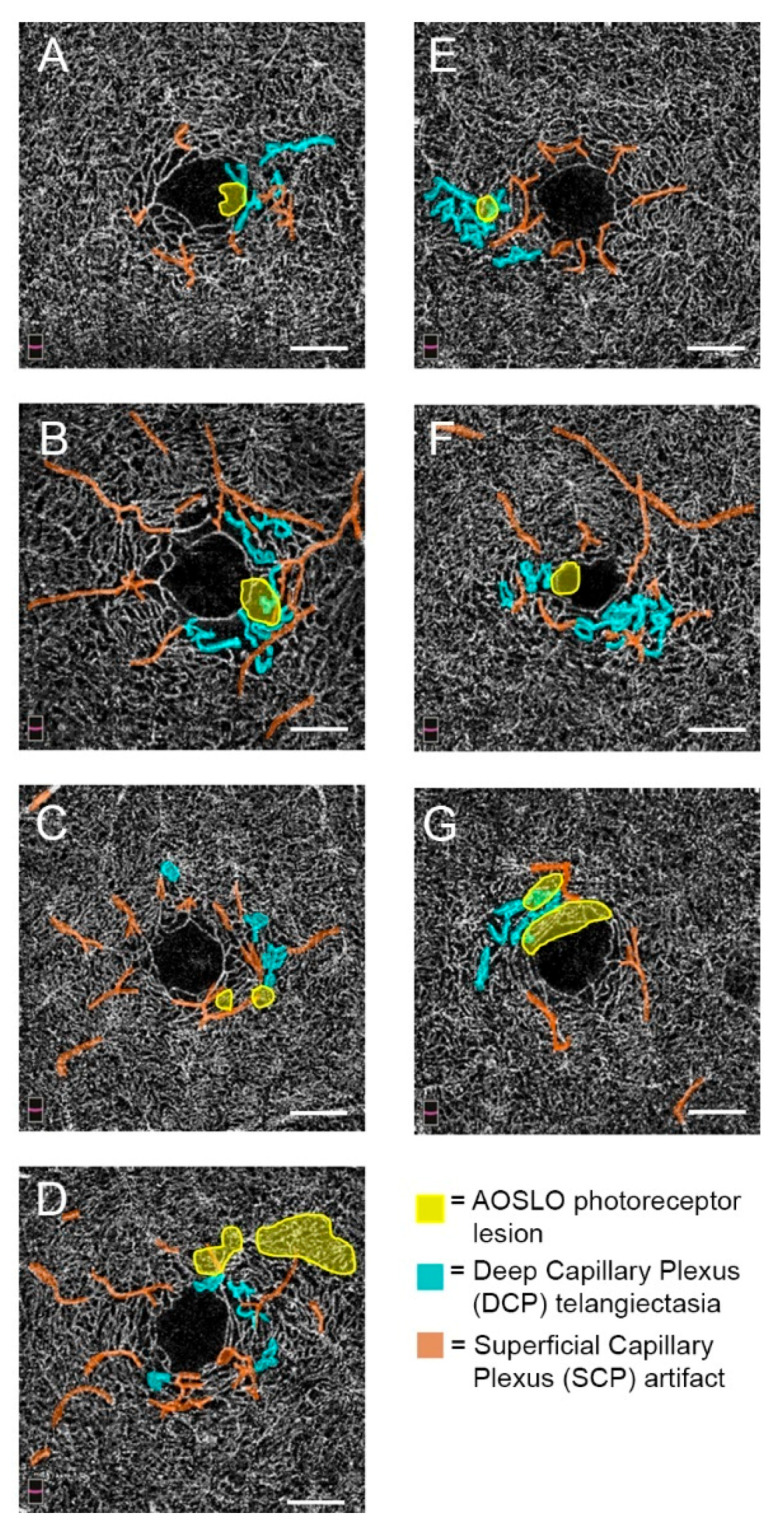
En-face optical coherence tomography angiography (OCTA) images of the deep capillary plexus (DCP) of MacTel eyes reveal areas of telangiectasias, that are in proximity to sites of AOSLO photoreceptor lesions. Subjects 1–7 (**A**–**G**). In each DCP slab, telangiectasias, shown in blue, are either adjacent to, or overlapping with, areas of photoreceptor lesions, shown in yellow. Superficial capillary plexus (SCP) artifacts are shown in orange. Scale bars = 500 μm.

All eyes had areas of DCP telangiectasias on OCTA. Of the parafoveal quadrants montaged using AOSLO, there were 10 quadrants of telangiectasias on OCTA. All of these were either adjacent or overlapping with ten total AOSLO photoreceptor lesions (Figure 2). Of note, subject 8 did not have an AOSLO lesion (Figure 1H) and was omitted from Figure 2; the montage was limited to the nasal quadrant due to images available for montaging—the only DCP telangiectatic vessels were in the opposite, nonmontaged, temporal quadrant. In comparison, subjects 1 through 7 all had lesions that occurred within quadrants with DCP telangiectasias. Specifically, three lesions were directly overlapping with DCP telangiectasias and five were abutting.

**Figure 3:**
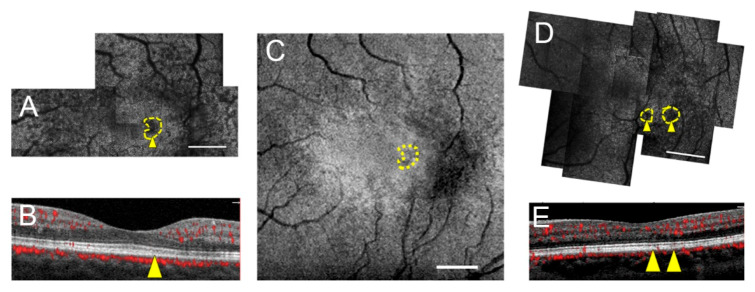
Three photoreceptor lesions correspond to both intact ellipsoid zone (EZ) and interdigitation zone (IZ) on spectral-domain OCT (SD-OCT) in two stage 0 MacTel eyes. Subjects 1 (left eye; **A**–**C**) and 3 (left eye; **D**,**E**). AOSLO montages with outlined, yellow lesion and yellow arrowhead (**A**,**D**) corresponding to accompanying normal SD-OCT cross-sections (**B**,**E**). (**C**) En-face structural OCT of the EZ with yellow outline of lesion identified on AOSLO in (**A**) reveals seemingly unremarkable structural appearance on OCT. Scale bars = 500 μm.

**Figure 4:**
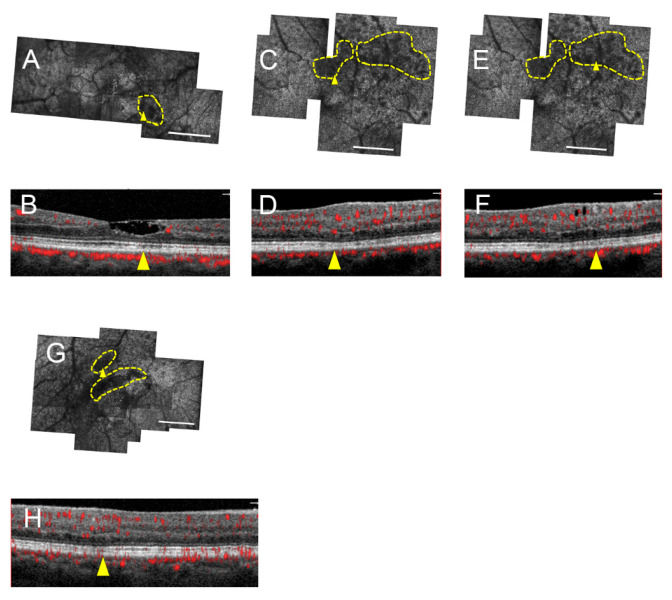
Comparison of AOSLO and SD-OCT cross-sections reveals photoreceptor lesions corresponding to intact EZ with either attenuated or absent IZ. Subjects 2 (left eye; **A**,**B**), 4 (left eye; **C**–**F**), and 7 (right eye; **G**,**H**). AOSLO montages with outlined, yellow lesion and yellow arrowhead (**A**,**C**,**E**,**G**) corresponding to accompanying SD-OCT cross-sections (**B**,**D**,**F**,**H**). IZ appears attenuated in (**B**) but absent in (**D**,**F**,**H**). Scale bars = 500 μm.

**Figure 5:**
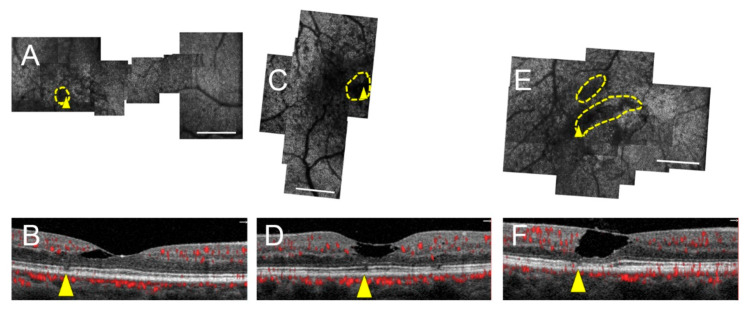
Photoreceptor lesions correspond to disrupted EZ in three stage 2 MacTel eyes. Subjects 5 (right eye; **A**,**B**), 6 (left eye; **C**,**D**), and 7 (right eye; **E**,**F**). AOSLO montages with outlined, yellow lesion and yellow arrowhead (**A**,**C**,**E**) corresponding to accompanying SD-OCT cross-sections (**B**,**D**,**F**). AOSLO lesions extend beyond areas of EZ loss (yellow arrowhead) on SD-OCT and partially correspond to the intact EZ. Scale bars = 500 μm.

SD-OCT evaluation of the areas of photoreceptor lesions revealed either corresponding intact EZ and IZ, intact EZ and attenuated/absent IZ, or disrupted EZ and IZ. The full area encompassing seven of ten lesions corresponded to intact EZ on SD-OCT (Figure 3 and Figure 4), four of which were in stage 0 eyes, two were in a stage 1 eye, and one was in a stage 2 eye. Partial areas of the remaining three lesions corresponded to intact EZ; these same lesions also partially corresponded to EZ loss (Figure 5). Of all photoreceptor lesions, there were three that corresponded to both intact EZ and IZ (Figure 3) and four that corresponded to the intact EZ and attenuated/absent IZ (Figure 4). There was not a single AOSLO lesion in which the entire area corresponded to EZ disruption. The external limiting membrane was intact in all eyes. Across all montages, excluding the central fovea and shadows from overlying vessels, there was only one area of EZ loss that occurred in an area of regular, hyperreflective cones on AOSLO.

## 4. Discussion

Among seven early stage MacTel eyes, we identified ten photoreceptor lesions consistent with zones of outer segment abnormalities on confocal AOSLO. We found that most of these lesions were either directly overlapping with or adjacent to DCP telangiectasias on OCTA, supporting our original hypothesis. Interestingly, most of these lesions corresponded to largely unremarkable SD-OCT appearance. While previous studies on MacTel have reported these dark regions on adaptive optics imaging that represent disturbances to the photoreceptors [14,15,17,26], our study establishes a topographic relationship between these areas and telangiectasias. Moreover, normal OCT appearance in the setting of these AOSLO photoreceptor lesions suggests that they may represent subclinical photoreceptor dysfunction.

We recognize from our previous work that some eyes with DCP telangiectasias in MacTel had no apparent EZ loss on SD-OCT [11]. To explain this phenomenon, we proposed the theory of “photoreceptors at risk” to explain the occurrence of telangiectasias in the setting of otherwise unremarkable photoreceptors. According to our hypothesis, these eyes could harbor photoreceptors with subclinical dysfunction (before the appearance of structural EZ loss), which may suppress normal antiangiogenic signaling leading to these abnormal DCP telangiectasias. In the current study, the lesions visualized on AOSLO may be representative of these “at-risk photoreceptors” in five of the eyes (Subjects 1–4, 7), three of which were classified as stage 0 (having no photoreceptor abnormalities on OCT). Seven photoreceptor lesions in these eyes, five of which were either abutting or overlapping with abnormal DCP telangiectasias, corresponded to an intact EZ on SD-OCT. While it is by no means definitive that these disrupted photoreceptors precede the DCP telangiectasias given the cross-sectional nature of this study, considering the extant understanding of the neurodegenerative pathogenesis of MacTel [4,6], these AOSLO lesions and our findings give credence to our theory of “photoreceptors at risk”.

Interestingly, three of the photoreceptor lesions corresponding to an intact EZ also corresponded to an intact IZ. In contrast, previous reports on MacTel using AOSLO and SD-OCT have found dark regions corresponding either to EZ loss [14,16,17] or an intact EZ with disrupted IZ [26]. We propose that these three photoreceptor lesions, corresponding to intact EZ and IZ in the setting of DCP telangiectasias, might represent the earliest evidence of subclinical photoreceptor dysfunction in MacTel. Moreover, the presence of an intact external limiting membrane offers an additional point of discussion. While the prevailing theory about MacTel pathogenesis currently posits that Muller cell dysfunction may be the primary cellular target [4,5], recent studies have shown that low serum serine levels are implicated in MacTel [26]. Serine is an important substrate in many metabolic pathways and its deficiency is associated with increased synthesis of atypical deoxysphingolipids, which are particularly toxic to neurons [26,27,28,29]. We postulate that an intact external limiting membrane on OCT with hyporeflective areas on AOSLO may be suggestive of early photoreceptor disruption (prior to Muller cell loss) as a consequence of serine deficiency.

The comparison between confocal AOSLO lesions with the structural integrity of OCT bands in each patient also reveals an interesting point about the use of the EZ band as a measure of photoreceptor function. Examining all of the lesions, it is evident that not all of them correspond to EZ loss. Also, for these lesions that did correspond to EZ loss, it appears that the AOSLO lesion extended further than the EZ loss on OCT. Conversely, there was also one area of normally reflective photoreceptors on confocal AOSLO that corresponded to EZ loss. These observations illustrate the nuances of the relationship between cone mosaic information on AOSLO and the EZ band on SD-OCT, a conclusion supported by the findings of Carroll and coworkers [16]. Furthermore, areas of nonwaveguiding cones do not necessarily mean photoreceptor death and may potentially be reversible in (1) the presence of an intact external limiting membrane [15,17], (2) in eyes where a disrupted IZ recovers [26], or (3) in eyes where remnant inner segments of these cones can be visualized on split-detector AOSLO [17]. For these reasons, all interpretations of AOSLO lesions should be treated with caution.

The findings of this qualitative, cross-sectional study should be considered with an understanding of its limitations. Our study included a small sample size and was limited to evaluating only the outer segment cones via confocal AOSLO. While the inner segment remnant cones were not studied due to lack of split-detector capabilities, our findings of photoreceptor disruption on confocal AOSLO in the vicinity of telangiectasias are no less valuable to our knowledge of the etiology of this disease. Thus, longitudinal studies investigating both the inner and outer segment cones with relation to telangiectasias over time is warranted to assess the recovery of photoreceptors as well as the evolution of DCP telangiectasias.

In summary, we explored the relationship between photoreceptor microstructure and pathologic telangiectasias in MacTel. Our findings of hyporeflective, nonwaveguiding photoreceptors adjacent to DCP telangiectasias suggest a topographic relationship between the neural and vascular components of this disease. Our work also illustrates the use of AOSLO for potentially defining the earliest pathologic photoreceptor change in the setting of normal SD-OCT.

## Figures and Tables

**Table 1:** Subject demographics, clinical characteristics, and clinical staging.

Subject	Age (Years)	Sex	Laterality	BCVA	OCT Stage
1	43	F	OS	20/25	0
2	68	M	OS	20/25	0
3	52	F	OS	20/25	0
4	60	F	OS	20/32	1
5	61	F	OD	20/20	1
6	59	F	OS	20/32	2
7	68	M	OD	20/20	2
8	48	M	OD	20/20	2

BCVA, best-corrected visual acuity; F, female; M, male; OCT, optical coherence tomography; OD, oculus dexter; OS, oculus sinister.

## Supplementary Materials

The following are available online at https://www.mdpi.com/2077-0383/9/10/3149/s1, Figure S1: Identification and exclusion of adaptive optics scanning laser ophthalmoscopy (AOSLO) lesions.

## References

[1] Issa P.C., Gillies M.C., Chew E.Y., Bird A.C., Heeren T.F., Peto T., Holz F.G., Scholl H.P. (2013). Macular telangiectasia type 2. Prog. Retin. Eye Res..

[2] Sallo F.B., Peto T., Egan C., Wolf-Schnurrbusch U.E.K., Clemons T.E., Gillies M.C., Pauleikhoff D., Rubin G.S., Chew E.Y., Bird A.C. (2012). The IS/OS Junction Layer in the Natural History of Type 2 Idiopathic Macular Telangiectasia. Investig. Ophthalmol. Vis. Sci..

[3] Gass J.D.M., Blodi B.A. (1993). Idiopathic juxtafoveolar retinal telangiectasis: Update of classification and follow-up study. Ophthalmology.

[4] Powner M.B., Gillies M.C., Zhu M., Vevis K., Hunyor A.P., Fruttiger M. (2013). Loss of Müller′s cells and photoreceptors in macular telangiectasia type 2. Ophthalmology.

[5] Powner M.B., Gillies M.C., Tretiach M., Scott A., Guymer R.H., Hageman G.S., Fruttiger M. (2010). Perifoveal Müller cell depletion in a case of macular telangiectasia type 2. Ophthalmology.

[6] Shen W., Fruttiger M., Zhu L., Chung S.H., Barnett N.L., Kirk J.K., Lee S., Coorey N.J., Killingsworth M., Sherman L.S. (2012). Conditional Müller cell ablation causes independent neuronal and vascular pathologies in a novel transgenic model. J. Neurosci..

[7] Spaide R.F., Klancnik J.M., Cooney M.J., Yannuzzi L.A., Balaratnasingam C., Dansingani K.K., Suzuki M. (2015). Volume-rendering optical coherence tomography angiography of macular telangiectasia type 2. Ophthalmology.

[8] Thorell M.R., Zhang Q., Huang Y., An L., Durbin M.K., Laron M., Sharma U., Stetson P.F., Gregori G., Wang R.K. (2014). Swept-source OCT angiography of macular telangiectasia type 2. Ophthalmic Surg. Lasers Imaging Retin..

[9] Gaudric A., Krivosic V., Tadayoni R. (2015). Outer retina capillary invasion and ellipsoid zone loss in macular telangiectasia type 2 imaged by optical coherence tomography angiography. Retina.

[10] Sallo F.B., Peto T., Egan C., Wolf-Schnurrbusch U.E.K., Clemons T.E., Gillies M.C., Pauleikhoff D., Rubin G.S., Chew E.Y., Bird A.C. (2012). “En face” OCT Imaging of the IS/OS Junction Line in Type 2 Idiopathic Macular Telangiectasia. Investig. Ophthalmol. Vis. Sci..

[11] Micevych P.S., Lee H.E., Fawzi A.A. (2019). Overlap between telangiectasia and photoreceptor loss increases with progression of macular telangiectasia type 2. PLoS ONE.

[12] Burns S.A., Elsner A.E., Sapoznik K.A., Warner R.L., Gast T.J. (2019). Adaptive optics imaging of the human retina. Prog. Retin. Eye Res..

[13] Litts K.M., Cooper R.F., Duncan J.L., Carroll J. (2017). Photoreceptor-based biomarkers in AOSLO retinal imaging. Investig. Ophthalmol. Vis. Sci..

[14] Ooto S., Hangai M., Takayama K., Arakawa N., Tsujikawa A., Koizumi H., Oshima S., Yoshimura N. (2011). High-resolution photoreceptor imaging in idiopathic macular telangiectasia type 2 using adaptive optics scanning laser ophthalmoscopy. Investig. Ophthalmol. Vis. Sci..

[15] Wang Q., Tuten W.S., Lujan B.J., Holland J., Bernstein P.S., Schwartz S.D., Duncan J.L., Roorda A. (2015). Adaptive optics microperimetry and OCT images show preserved function and recovery of cone visibility in macular telangiectasia type 2 retinal lesions. Investig. Ophthalmol. Vis. Sci..

[16] Scoles D., Flatter J.A., Cooper R.F., Langlo C.S., Robison S., Neitz M., Weinberg D.V., Pennesi M.E., Han D.P., Dubra A. (2016). Assessing photoreceptor structure associated with ellipsoid zone disruptions visualized with optical coherence tomography. Retina.

[17] Litts K.M., Okada M., Heeren T.F., Kalitzeos A., Rocco V., Mastey R.R., Singh N., Kane T., Kasilian M., Fruttiger M. (2020). Longitudinal assessment of remnant foveal cone structure in a case series of early macular telangiectasia type 2. Transl. Vis. Sci. Technol..

[18] Khodabande A., Roohipoor R., Zamani J., Mirghorbani M., Zolfaghari H., Karami S., Modjtahedi B.S. (2019). Management of idiopathic macular telangiectasia type 2. Ophthalmol. Ther..

[19] Chew E.Y., Clemons T.E., Jaffe G.J., Johnson C.A., Farsiu S., Lad E.M., Guymer R., Rosenfeld P., Hubschman J.-P., Constable I. (2019). Effect of ciliary neurotrophic factor on retinal neurodegeneration in patients with macular telangiectasia type 2: A randomized clinical trial. Ophthalmology.

[20] Chew E.Y., Peto T., Clemons T.E., Pauleikhoff D., Sallo F.B., Heeren T., Egan C.A., Charbel Issa P., Balaskas K., Bird A.C. (2019). A New Classification for Macular Telangiectasia type 2 based on multi-modal imaging. Investig. Ophthalmol. Vis. Sci..

[21] Jia Y., Tan O., Tokayer J., Potsaid B., Wang Y., Liu J.J., Kraus M.F., Subhash H., Fujimoto J.G., Hornegger J. (2012). Split-spectrum amplitude-decorrelation angiography with optical coherence tomography. Opt. Express.

[22] Dubra A., Sulai Y. (2011). Reflective afocal broadband adaptive optics scanning ophthalmoscope. Biomed. Opt. Express.

[23] Onishi A.C., Roberts P.K., Jampol L.M., Nesper P.L., Fawzi A.A. (2019). Characterization and correlation of “Jampol dots” on adaptive optics with foveal granularity on conventional fundus imaging. Retina.

[24] Cooper R.F., Sulai Y.N., Dubis A.M., Chui T.Y., Rosen R.B., Michaelides M., Dubra A., Carroll J. (2016). Effects of intraframe distortion on measures of cone mosaic geometry from adaptive optics scanning light ophthalmoscopy. Transl. Vis. Sci. Technol..

[25] Scoles D., Sulai Y.N., Langlo C.S., Fishman G.A., Curcio C.A., Carroll J., Dubra A. (2014). In vivo imaging of human cone photoreceptor inner segments. Investig. Ophthalmol. Vis. Sci..

[26] Gantner M.L., Eade K., Wallace M., Handzlik M.K., Fallon R., Trombley J., Bonelli R., Giles S., Harkins-Perry S., Heeren T.F. (2019). Serine and lipid metabolism in macular disease and peripheral neuropathy. N. Engl. J. Med..

[27] Zhang T., Gillies M.C., Madigan M.C., Shen W., Du J., Grünert U., Zhou F., Yam M., Zhu L. (2018). Disruption of de novo serine synthesis in müller cells induced mitochondrial dysfunction and aggravated oxidative damage. Mol. Neurobiol..

[28] Scerri T.S., Quaglieri A., Cai C., Zernant J., Matsunami N., Baird L., Scheppke L., Bonelli R., Yannuzzi L.A., Friedlander M. (2017). Genome-wide analyses identify common variants associated with macular telangiectasia type 2. Nat. Genet..

[29] Zhang T., Zhu L., Madigan M.C., Liu W., Shen W., Cherepanoff S., Zhou F., Zeng S., Du J., Gillies M.C. (2019). Human macular Müller cells rely more on serine biosynthesis to combat oxidative stress than those from the periphery. Elife.

