# Supplementary material for: Topographic Relationship between Telangiectasia and Cone Mosaic Disruption in Macular Telangiectasia Type 2
Source: J Clin Med. 2020 Sep 29;9(10):3149. doi: 10.3390/jcm9103149 (PMC7601362; doi:10.3390/jcm9103149)
Supplement: Supplementary file 1 [file jcm-09-03149-s001.pdf]

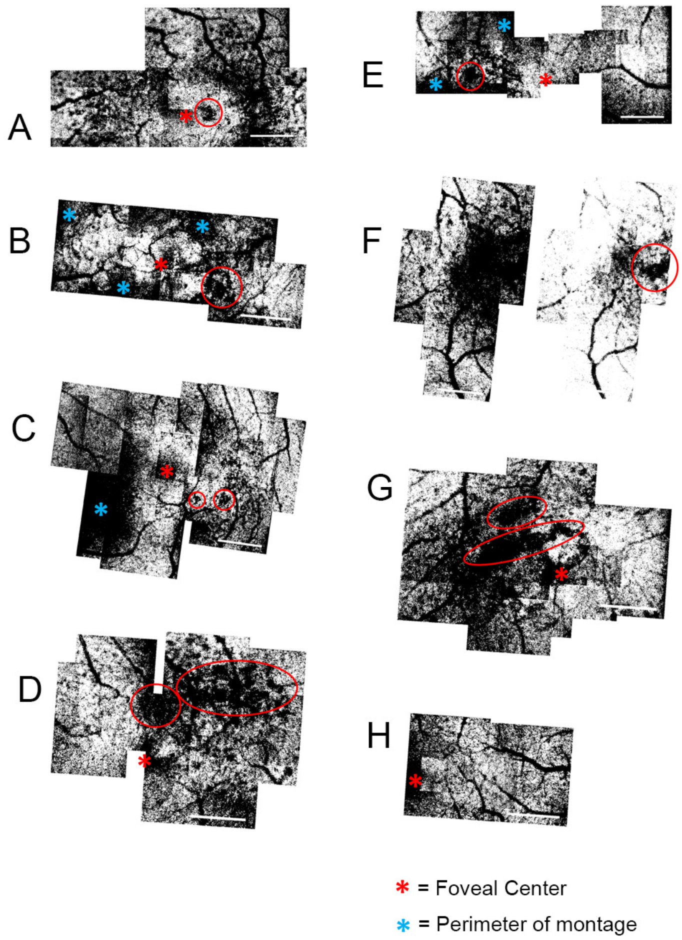

Figure S1. Identification and exclusion of adaptive optics scanning laser ophthalmoscopy (AOSLO) lesions. Subjects 1-8 (A–H). Lesions (red circle) included discrete hypo-reflective areas of at least 100 μm abutting hyper-reflective areas. The foveal center, montage perimeters, and vessel artifacts were excluded. Scale bars = 500 μm.
